# Interrogating artificial agency

**DOI:** 10.3389/fpsyg.2024.1449320

**Published:** 2025-01-17

**Authors:** Hong Yu Wong

**Affiliations:** Department of Philosophy and Centre for Integrative Neuroscience, University of Tübingen, Tübingen, Germany

**Keywords:** artificial intelligence, agency, action, Turing test, ChatGPT

## Abstract

Can artificial systems act? In the literature we find two camps: sceptics and believers. But the issue of whether artificial systems can act and, if so, how, has not been systematically discussed. This is a foundational question for the philosophy of AI. I sketch a methodological approach to investigating the agency of artificial systems from architectural and behavioural perspectives.

## 1 Introduction: the phenomenon in question

Artificial intelligence (AI) seems to be all the rage now. AI is almost never out of the news cycle—whether it concerns accidents involving self-driving cars, doomsday prophecies about AI taking over the world, AI causing workers to lose their jobs or freeing everyone from laborious tasks (on alternate days), and the latest tricks that the newest version of Large Language Models (LLMs) can perform. Notice that AI appears to be doing things in all these newsworthy examples. Further, AI is not only taken to do things—AI’s agency seems to be more or less taken for granted. It is because AI can do things that it can achieve all these effects. AI is driving cars; AI is achieving superintelligence and world domination; AI is taking the jobs of workers and causing unemployment or saving humans from mind-numbing work and so creating utopia; LLMs are learning how to answer questions in basic arithmetic better.

Some sceptics dismiss all this talk of AI doing things as merely a Façon de Parler. Their suggestion is that none of this should be taken seriously. To do or not to do; who cares? They might say. This talk of AI doing things is just like talk of volcanoes destroying villages or making the land fertile, scarecrows scaring off birds, detergent cleaning stains, or sunflowers turning to the sun. It is just a manner of speaking; nothing hangs on it. It is quite unlike people doing things—or for that matter, bats, bees, or buffaloes doing things. Is it fair to exclude artificial systems from the realm of agency?

This sceptical or, more appropriately, dismissive, position is counterbalanced by the apparent nonchalance with which AI researchers attribute agency to AI. The Bible of AI, Russell and Norvig’s (2020) classic textbook, Artificial Intelligence: A Modern Approach, begins with the entirely undefended premise that artificial systems are agents. They tell us that their “main unifying theme is the idea of an intelligent agent” (their emphasis) and immediately proceed to define AI as “the study of agents that receive percepts from the environment and perform actions.” They continue, “Each such agent implements a function that maps percept sequences to actions, and we cover different ways to represent these functions, such as reactive agents, real-time planners, decision-theoretic systems, and deep learning systems.” (Russell and Norvig, 2020). If so, then AI systems are agents and can act. This is not just a façon de parler as the whole project of AI is presented as being premised on the ability of AI to actuate and do things.

We find in the literature various reasons, ethical and practical, for taking artificial agents as agents, but the question whether or how artificial agency is possible is not even raised. It is simply assumed that artificial systems are agents that can act (e.g., Ågerfalk, 2020; Cohen and Levesque, 1995; Coeckelbergh, 2015; Russell and Norvig, 2020; Floridi, 2023). Is this tenable? Rather surprisingly, this nonchalance toward agency contrasts quite strikingly with the attitude of many AI researchers toward whether AI has genuine intelligence or understanding. They seem to be much more circumspect on the latter (e.g., Russell and Norvig, 2020 and the commentaries on Searle, 1980). This may be due to contingent sociological factors related to the prominence of Searle’s (1980) Chinese Room argument in the history of AI. But if AI researchers would stop to reflect for a moment, it would seem that the issues about agency and intelligence are not so very different.

Whither artificial agency? How do we decide whether the sceptics or the believers are correct? And is the disagreement even significant? If so, what hangs on it? My aim in this essay is to reflect on the possibility of artificial agency from first principles.

## 2 How should we pose the problem of artificial agency?

How can we go about interrogating artificial systems about their agency? The usual way in the philosophy of action is to consider whether some candidate behaviour should be considered an action or not. This is known as the problem of action (e.g., Frankfurt, 1978; Velleman, 2014; Railton, 2017).^1^ Here the question is how we can explicate the contrast between something active that the agent does, as opposed to something passive that merely happens to her. This is an intuitive distinction that captures both the phenomenology of acting, the moral significance of some behaviour being agentive or not (in/voluntary), and aligns with the scientific idea that there is a real difference between some behaviour being an action or not (Frankfurt, 1978; Jeannerod, 1997).

The contrast posed applies equally to bodily and mental actions. In the case of bodily action, we can contrast active self-movement, e.g., raising one’s hand to greet someone, as opposed to one’s hand passively rising from a Transcranial Magnetic Stimulation (TMS) pulse; or my kicking from my knee versus a kicking due to the patellar reflex. In the case of mental action, we can, e.g., contrast a case of deliberately thinking through a problem and arriving at the solution through reasoning, as opposed to the solution coming unbidden, when mind wandering. Philosophers of action try to explicate these intuitive contrasts through providing a theory of action, bodily and/or mental. Would such an approach work in the case of AI?

If we were looking to apply this approach to AI, the task would be to distinguish cases of an AI system’s doing something actively as opposed to something merely happening to it. The question would then be to ask which processes in AI systems look like cases of processes where the system is active. No doubt we can imagine such cases and would be able to draw the active/passive distinction in such a (counterfactual) scenario.^2^ But to be able to conceive of an AI as possibly acting is not to conceive of how it could act.

Let me observe that there are actually two problems in the vicinity, rather than the single one that is typically at the focus of the philosophy of action:

- The problem of action: When is a stretch of behaviour an exercise of the system’s agency as opposed to something that merely happens to the system?^3^
- The problem of agenthood: Can the system act? Or: Is the system an agent?

Questions A and B are related but are distinct. A presupposes a positive answer to B. If the system cannot act, then no stretch of its behaviour can be an exercise of agency. In this sense question B is prior to question A. However B is not often addressed. As a matter of fact, philosophers have focused almost entirely on answering question A for what they have considered the core cases of healthy adult human agency. Since the status of healthy adult humans as agents is never really in question,^4^ philosophers consider question B to have been answered affirmatively. Philosophers have thus immediately focused on A. Though there is little discussion of the relation between questions A and B, because they are not usually explicitly distinguished, the starting point of any discussion of agency would appear to depend on the agentive status of the system in question. In philosophy of action, discussion has overwhelmingly focused on healthy adult human action. Even dissenters mainly discuss cases of healthy adult human action that do not fit the “standard story” (Velleman, 2014), such as subintentional actions (O’Shaughnessy, 2008; Steward, 2009) or “arational” expressive actions (Hursthouse, 1991; Müller and Wong, 2024). More recently there has been discussion of how agency has evolved and how it develops (e.g., Sterelny, 1995; Nudds and Hurley, 2006; Burge, 2009; Steward, 2009; Butterfill, 2020; Tomasello, 2022). Consequently, the question of whether some organism is an agent or not is prioritised to the degree that there is uncertainty about its agentive capacities. Previously the prevailing view was that language was a prerequisite for action; because action requires reason, and reason requires language (see, e.g., McDowell, 1996). But this chauvinistic view of taking the conditions on healthy adult human agency as the conditions on agency simpliciter have, of late, come under serious fire.

The thought—never explicitly articulated—was in all likelihood that insofar as agency was the province only of healthy adult humans, their status as agents was unquestionable—and thus question A was naturally the focus of reflection. However, once we venture beyond the most obvious case of agents, matters get more complex. Even the case of human beings is complicated. When does an infant become an agent? Or are they already agents, but simply grow in the complexity of control structures that guide action as they age? This requires thinking through question B for human children and infants. As for non-human animals, we have always been more willing to attribute the gift of agency to those animals closest to us, and increasingly uncomfortable with attributions the further from us the creatures are phylogenetically. But since the recognition of the importance of animal rights, these questions have come to the forefront. Thus question B becomes vital. Question B must then be addressed first before question A can be approached. Of course, in order to answer B, some conception of possible answers to A for at least a significant part of the core behavioural repertoire of the candidate system is required. AI and the agentive status of artificial systems puts further pressure on the traditional philosophical view. Once again question B needs to be answered. So it would seem that in cases where we are unsure of the agentive status of the target system for analysis, question B must be addressed before serious research on A can begin. Thus in a sense question B—the question of whether a system is an agent is prior. If we return to Russell and Norvig’s (2020) definition of AI as “the study of [intelligent] agents,” then what I am adding here is that we urgently need to answer question B for AI. Only then can we answer question A for AI, if at all.^5^

## 3 Approaches to artificial agency

How does one go about answering the question which artificial systems are agents? We must reflect methodologically on this.

One view is that digital agency, the agency of informational systems, is parasitic on human agency in a way that human agency is constitutive and required for it (Ågerfalk, 2020). This is akin to Searle’s (1980) suggestion that AI cannot have genuine but only derivative intentionality. Agency in this sense is the “capability of machines to act autonomously, but on behalf of humans, organisations and institutions” (Ågerfalk, 2020, p. 5). Even if artificial agency is parasitic on human agency in some sense, this still means that artificial agents can act. So we must face the question of what artificial agency is head on.

There are objections to artificial agency deriving from the thought that genuine agency requires genuine understanding or some form of mindedness, which artificial systems do not have (Searle, 1980). The idea is that agency requires understanding, because standard models of agency require that actions are on the basis of reasons (Davidson, 2001; Railton, 2017). A system cannot have reasons in this sense without some intelligence or understanding. On this picture, intelligence then is a requirement on agency. The acknowledgement of primitive agency opens up potential responses here for the possibility of artificial agency, because primitive actions do not necessarily require reason or mindedness (Burge, 2009).

The question of artificial agency opens certain avenues for investigation here where the constitutive questions about agency can come apart from questions about evolutionary history, since new systems can be designed which have architectures that have no biological precedent. The question of artificial agency thus offers a unique opportunity for a case study where we can bring together different aspects of action theory and to repose some key questions about action in a new way.

We will need to ask questions about the epistemology of action—when can we see action? When can we ascribe agency?—and questions about what underlying architectures of systems we can acknowledge as having agentive capacities, balancing these considerations in giving an account of artificial agency. These questions are especially pertinent now with the rise of generative artificial intelligence and widespread use in the populace of such LLMs, such as ChatGPT (Schwitzgebel et al., 2024). Given that there is widespread linguistic and communicative interactions with such systems, our interactions could be argued to presuppose linguistic and communicative agency on the part of such AI systems.

A conceptualization of what is happening in these cases is required. How do we go about this? I suggest that there are two broad approaches or perspectives to this question which dominate research on agency. While they have not been explicitly distinguished and identified as such, they have been influential.^6^

### 3.1 Architectural versus behavioural approaches to agency

The two broad perspectives to agency are what we might call an “architectural” as opposed to a “behavioural” approach. The labels reflect the core idea behind the approach. The architectural approach emphasises that certain kinds of underlying mechanism or organisational structure of the system is required for the capacity to act. We can think of the approach here as like that of a physiologist. Agency is one of the proper functions of the organism and the physiologist aims to understand the underlying mechanisms which underpin this capacity.^7^

In contrast, the behavioural approach stresses not internal structure or mechanism, but rather behaviour. Does the system look like it is acting? Can the system be attributed agency based on observing its behaviour? Or even more strongly: can the behaviour of the system only be made sense of by attributing agency? We can think of the approach here as like that of a field biologist or anthropologist in a “first contact” situation, where they are encountering a new species or a new system for the first time. They cannot analyse the candidate system by understanding its internal structure and dynamics. All they have is behavioural observation—and perhaps not only passive observation but also interaction with the system as well.

Let me delve into each of the approaches in more depth below.

### 3.2 Architectural approaches

The architectural approach is the default position of most action researchers, both philosophical and scientific. Both philosophically and scientifically the approach stems from identifying some class of systems as paragon agents, then extrapolating and abstracting from details of the capacities and internal architecture of such systems to arrive at accounts of agency and action. We can summarise the architectural approach with the following sequence of questions which characterise the approach:

**Architectural approach: leading questions**

A1. Which systems are genuinely agentive?

A2. What architecture do they have?

A3. Can we extrapolate insights from their architecture for understanding agency?

As I sketched earlier, the approach is like that of a physiologist’s. Agency is one of the proper functions of the organism/system and the physiologist aims to understand the function and underpinnings of agency. Though it may not be obvious at first sight, the architectural approach covers a wide range of positions about action and agency which in the standard debate in action theory are seen as competitors (Ferrero, 2022). Vastly different theories of action, for example, ranging from the hermeneutic, anti-causalist theories, such as Anscombe’s (1957) Intention, to biologically-inspired accounts of agency that are sensitive to considerations about the evolution of agency (e.g., Gallistel, 1980; Burge, 2009; Tomasello, 2022), all fall under the architectural approach. Allow me to explain by walking through how these different approaches are all architectural.

*Philosophical approaches*: let us begin from what might be called the “core tenet” of philosophical action theory—agents act on the basis of reasons and these reasons explain their actions. This might seem not to be an architectural conception of action first, but a constitutive account of action. The standard package for introducing the core tenet in philosophical action theory includes accepting Anscombe (1957) “Why?”-question as criterial for intentional action. According to Anscombe (1957), an action is intentional if and only if the agent is in a position to answer why the action was done from their first-person perspective. The agent naturally does not have to have been rehearsing the reasons for acting as she was acting. The reasoning just needs to be available to her and the possible “Why?”-questions and answers reflect the teleological structure inherent in the action (Vogler, 2002). On this picture, human beings are the only genuine agents (A1), because they have the capacity of reason (A2), and they are agents because they can do things for reasons (A3). This basic picture of intentional action is accepted by most philosophers working on action, whether or not they are causal theorists of action (e.g., Anscombe, 1957; Davidson, 2001; Vogler, 2002). The causal theorists would supplement what we have described of Anscombe’s “Why?”-question apparatus with various motivational states which capture or encode the agent’s reasons for acting, such as belief-desire pairs (Davidson, 2001), intentions (Searle, 2012), plans (Bratman, 1987) or other higher-order states (Velleman, 1992). The architectural constraint here is the idea that agents have to have the faculty of reason. If we move beyond the classical philosophical positions on action around Anscombe, Davidson, and their supporters to more outré positions in the philosophy of action, we also find the architectural approach represented strongly. For example, if we consider more empirically-informed and oriented work in the philosophy of action, then we find an emphasis on the motor system (Pacherie, 2008; Butterfill, 2020; Wong, 2018), affective system (Railton, 2017) or executive control (Buehler, 2022; Sripada, 2021) as crucial.

*Physiological approaches*: in physiology, the distinction between the central and peripheral nervous system in the control of behaviour is vital for understanding agency and action (Gallistel, 1980; Tomasello, 2022). Here the distinction between action and mere behaviour is understood by way of the physiological distinction between action versus reflex, where actions are seen as flexible goal-directed behaviour on the part of the whole organism, whereas reflexes are inflexible. Action is seen as underpinned by control mechanisms in the central nervous system, whereas reflexes are peripherally controlled. This way of thinking about action has also influenced philosophical action theory (Frankfurt, 1978; Burge, 2009; Wong, 2018). For example, in his discussion of primitive agency, Burge (2009) proposes that even certain unicellular organisms can be understood as agents, where “… the relevant notion of action is grounded in *functioning*, *coordinated behavior* by the *whole organism*, issuing from the individual’s central behavioral capacities, not purely from sub-systems. Coordination is meant to imply that the behavior must issue from central capacities, in effect coordinating sub-systems, or coordinating central capacities with their peripheral realizations.” (emphases in the original). Here Burge abstracts away from the central/peripheral distinction about nervous systems of complex animals and applies something like it to unicellular organisms. He understands coordination and control of behaviour as coming down to central control over peripheral mechanisms. This is clearly an architectural conception of agency.

*Biologically-inspired approaches*: Yet another architectural approach is the biologically-inspired approaches deriving from the work of Maturana and Varela (1980) on autopoesis. On this picture, the key to agency is life. Living organisms are agents. Here the focus is explicitly on agents rather than actions, in contrast to most philosophical action theory. This is partly because the autopoetic approaches focus on how the system as a whole relates to its environment, emphasising the system’s biological autonomy. “Biological autonomy comprises … basic autonomy through material self-constitution and adaptivity through interaction with the environment. In higher organisms, these two aspects are supported by two different subsystems: metabolism on the one hand and the nervous system on the other, the latter giving rise to cognition as opposed to mere life as being provided by metabolism” (Meincke, 2018, explaining Moreno and Etxeberria, 2005). The goals of the system are its own goals because they arise from the contribution those goals make to adapting to keep the system alive in its environment. Goals thus arise from a contribution to metabolism. Taking inspiration from this biological approach, one can extrapolate from the organisation principles of self-constitution (metabolism and homeostasis), adaptivity, and coupling required for organism-environment interactions to more abstract principles needed for the organisation of a life-like system. Thus Barandiaran et al. (2009) propose that an “agent is an autonomous organisation capable of adaptively regulating its coupling with the environment according to the norms established by its own viability conditions.” Overall the idea is that in order to be agents, systems must have their own goals in some sense, which arise from their contribution to the system’s own adaptive self-maintenance in its environment. Here the architectural idea is that agentive systems must be living systems or life-like systems. The architectural requirements on agency are thus those on living or life-like systems.

To summarise: despite the dramatic differences in what architecture the views discussed above take to be vital for agency and action, all of them posit some internal architecture underpinning flexible, adaptive, rational capacities of movement, which are seen as vital for being an agent.^8^

### 3.3 Behavioural approaches

Another important approach to agency comes from a behavioural perspective. Instead of focusing on internal architecture this perspective focuses on the interpretability of candidate systems as agents based on observing the system’s behaviour. We can see this method as a fieldwork approach to agency, where one is in the role of a field biologist or an anthropologist—as opposed to that of a physiologist (as in the architectural approach).

The first role of the researcher is, thus, to closely observe the behaviour of the candidate system. While observing the system, the researcher would have to ask: Is there the kind of behavioural flexibility and adaptivity that would require the attribution of agency to the candidate system? Let us consider the circumstances under which such a question might arise. This question would arise when there are stretches of behaviour the observer can better understand by drawing a distinction between the candidate system seemingly doing things as opposed to things merely happening to it—that is, drawing a distinction between a system’s acting as opposed to not acting.

This takes us back to our discussion in section 2 about how we should pose the problem of artificial agency. I argued that question A, the problem of action, is parasitic in a way on question B, the problem of agenthood. We see a similar situation reflected here in fieldwork. It is possible to focus in fieldwork on drawing the distinction between cases of action as opposed to mere behaviour based on observation. But this is a difficult project, since it is not easy to draw that distinction based on observation of behaviour since they may be sometimes indistinguishable from the outside (Smith, 2010). Interestingly, even though it is not straightforward to decide whether any individual stretch of behaviour is an action or not, this difficulty does not necessarily translate to the decision of whether a system is agentive or not. Even if one’s judgement may be uncertain, we seem to have more confidence in judging whether a system is an agent or not.

Having made these preliminary observations, let us now consider the leading questions of this behavioural approach to agency are:

**Behavioural approach: leading questions**

B1. Does the candidate system behave like it is acting?

B2. Can we appropriately deploy the intentional stance (a stance where the observer has to treat the observed system as if it were an intentional system; Dennett, 1989) to understand the system?

Question B1 is exactly Turing’s approach. One way to circumvent the difficulties I outlined above in introducing the fieldwork approach is to take the kind of operational approach Turing (1950) recommended in his famous “imitation game”—or the Turing test, as we now call it. Turing wished to address the question of when AI could think and was frustrated with existing approaches that were either semantic or architectural. Turing suggested an innovative behavioural criterion instead. His proposal was that if a human observer could not distinguish between conversing with an AI as opposed to another human being, then the AI should count as passing the test for thinking. Turing chose conversation as the domain of evaluation. Conversation would appear to be a good way to evaluate the capacity of thought—that is what we do in our everyday lives and in job interviews. Whatever capacities of thought that are at the core of its explaining intelligence would appear to be expressed in conversation and to be dissociable from its role in guiding movement. After all, non-linguistic animals can move but not converse. To further ensure that the test does not succumb immediately to anthropomorphic prejudices, such as sounding a certain way a human might sound or having a human-like body, the conversation should be conducted over a computer terminal to remove these elements.

When a human observer cannot distinguish between conversing with an AI as opposed to another human being over a computer terminal, that would mean that that AI would be behaviourally indistinguishable from a human responder for a human interrogator. So the test is a test of behavioural indistinguishability from a human agent in conversation. We can take the Turing test effectively as a test for agency as well. Ordinarily, if a system would count as having the capacity for thought, then it would have the capacity to act, since thinking is a form of agency (even if one does not think that all thoughts are actions, e.g., when one is mind wandering).

One question about Turing’s approach is why behavioural indistinguishability from a human respondent in a conversational context should be the operational criterion for being a thinker. It is certainly stronger than what would be needed to establish being an agent. One might think that there are a whole series of different tests of behavioural indistinguishability from target systems which count as paradigm agents—conversation is one domain which could provide a criterion, another domain might be movement. And, of course, within movement we can distinguish between very many different classes of movement (e.g., considering whether certain forms of taxes of single-celled organisms might count as action or not; Burge, 2009). But why should we privilege behavioural indistinguishability from some paradigm agent as a criterion for being an agent simpliciter? Surely this is a methodological bias that would count against possible agents that behave rather differently from the systems which have been recognised as paradigm agents.

Turning to B2: one way to get around the worry above is to take a more abstract behavioural approach based on seeing whether making sense of the observed behaviour would require attributing internal states that are characteristic of those of agentive systems. This is Dennett’s approach in terms of taking the intentional stance (Dennett, 1989). His thought is that whether a system is an agentive system comes down to whether we can appropriately deploy the intentional stance to understand the system. That is, do we require attributing intentional attitudes, such as beliefs, desires, and other mental states, to the system to make sense of its behaviour? On Dennett’s view, the truth of whether a system is an intentional system—that is, an agentive system—comes down to whether we can appropriately deploy the intentional stance to understand the system.

What is it to deploy the intentional stance? Dennett introduces the intentional stance by way of contrasting it with other stances: the physical stance and the design stance. The physical stance is the stance an observer takes to a system when trying to understand it according to the laws of physics and initial conditions. But maybe not all systems are best understood through taking a physical stance toward them. Consider a thermostat. We can take the physical stance toward it and would be able to describe how the circuit would be broken or completed depending on the temperature. But we would miss how it is designed to do that based on temperature so as to regulate the temperature in a room. Thus in understanding a technological artefact, like a thermostat or an automobile or a chair, we need to take the design stance to understand the system. In so doing, we are understanding the system by trying to make sense of what it is designed for—its purpose and its function. I have been talking of the design stance as applying to artefacts but it could equally be applied to biological systems which are “designed” by evolution to have a proper function and purpose. So, for example, to understand the function of the otolithic organs in the inner ear we cannot only look at them as physical systems but have to understand their role in the vestibular system in order to understand what they do. In fact, in understanding a system from the design stance, we need not necessarily understand the system from a physical stance. The intentional stance is yet another stance an observer can take toward a system. When neither the physical nor the design stance is sufficient to make sense of the behaviour of a system, we may need to attribute to the system certain intentional states, such as perceptual, cognitive, and conative states in order to make sense of how the system is behaving. In that case, we attribute the system various mental states, like perception, belief, desire, emotion, and memory, to take some examples. And, in virtue of the attribution of these states, we can understand and make predictions about the behaviour of the system that we could not before, by only taking the physical and/or the design stance. In taking such a stance to make sense of the behaviour of a system we do not need to know anything about its internal material constitution or its design. For example, in observing a mouse run away from a cat, I can make sense of it by ascribing to the mouse a perception of the cat and fear of the cat. Or if I know that Krisztina likes Handel, I can predict that when the opera puts on Orlando, Krisztina will buy tickets and attend the opera. I do not need to know about her as a biological system or her physical constitution to predict this. Dennett emphasises that even if we had a Martian scientist, who was like a Laplacian Demon in being able to calculate everything from a physical stance, the Martian would in fact be missing certain patterns—“real patterns” (Dennett, 1989)—at the intentional level of generalisation which would provide for compacter and better generalisations of the system’s behaviour than predictions from its physical properties.^9^

These views canvassed above provide a behavioural perspective on the attribution of agency: either behavioural indistinguishability from a paradigm agentive system (the Turing test) or deploying the intentional stance (Dennett). These are approaches which attribute agency without a commitment to the internal architecture of the candidate system.

## 4 Issues with each perspective

Each perspective is insightful. But, unfortunately, neither is sufficient on its own for facing the problem of determining whether artificial systems can be agents. Let us examine issues with each perspective.

The main issue with the architectural perspective has to do with its chauvinism toward certain architectures. Because of its historical association with classical philosophical or physiological views, there is an overwhelming emphasis on either architectures associated with rationality and language or with the physiological idea of the central/peripheral nervous system distinction. This contrasts with more distributed architectures—and *a priori* other possibilities we have not even begun to consider. Here we have to ask whether we already know that certain other architectures simply cannot sustain agency. Are there a priori reasons why certain architectures cannot sustain agency? We would need such considerations to exclude them.

The main issue with the behavioural perspective has to do with over-attribution. A familiar result, dating back to Heider and Simmel’s (1944) demonstration that we are willing to attribute agency even to apparently moving two dimensional geometric figures which look nothing like normal agentive systems, is that we are prone to over-attribute agency. The behavioural perspective can respond by finessing the agency attributions and adding further conditions. As I have already pointed out, behavioural indistinguishability from some paradigm agentive system as a criterion also suffers from a kind of chauvinism—who is to say that only systems which behave like them can be agentive? The different behavioural stance approaches try to get around this by setting appropriateness conditions on agency ascription: for Dennett’s intentional stance it comes from how the attribution of intentional states that makes sense of the system’s behaviour without which certain “real patterns” would be lost. Even if the intentional stance can impose conditions strict enough to discipline our tendencies toward over-attribution, a deeper problem remains: What is the basis of the agency attribution? How can it be made without an architectural ground? Even if architectural grounds are not initially required, if it were discovered that there are no plausible architectural grounds for the attribution, surely the attribution of agency would be withdrawn even if strict behavioural criteria were fulfilled.

## 5 Morals moving forward

The lesson of our discussion so far is that any way of moving forward on evaluating the agentive status of a system must simultaneously draw on both architectural and behavioural considerations. But what does this mean practically for the evaluation of the agentive status of AI systems? It will be useful try to see what morals we have moving forward by briefly looking at an AI system.

Let us consider the large language models (LLMs) that are highly popular nowadays, such as ChatGPT. Other examples of such LLMs are Gemini, Claude, and Llama. The points I make below also apply to them. ChatGPT is a chatbot released in late 2022 by the company Open AI. It allows users to converse with it in different languages, where user prompts and replies function as a continuous conversational context and can be used to shape ChatGPT’s responses. The architecture behind ChatGPT involves OpenAI’s generative pre-trained transformer (GPT) models and further fine-tuning for conversation is done using supervised learning and reinforcement learning with human feedback.

In conversational contexts, what ChatGPT and other LLMs effectively do is to predict the most likely next text token, and so have been dismissed as being “stochastic parrots” (Bender et al., 2021). Despite this, ChatGPT has remarkable versatility and can be prompted to do a huge range of things, including writing and editing code, composing stories, writing essays, answering exams of different sorts. Many users use it as a research assistant for summarising texts or translating texts in other languages. Some people use it for brainstorming; others use it like a psychotherapist. Thus, ChatGPT almost functions like a kind of personal assistant. Certainly, personal assistants have agency and can do things, which is why people employ them. So does ChatGPT have agency too? Or is it more like a tool, like a hammer or a thesaurus, which one can use to do things, but has no agency of its own?

How does ChatGPT fare with respect to the two perspectives with which we sketched above for studying agency? From the behavioural perspective, the Turing test has loomed large. Many researchers now think that ChatGPT has passed the Turing test, though no official tests have been done since the Loebner Prize was last run in 2019. A recent news feature in the leading science journal Nature was titled “ChatGPT broke the Turing test” (Biever, 2023). When interviewed for this news feature, some leading researchers, like Chollet, claim that they can tell that from the conversational responses whether it is an LLM or a human. Note that this does not show that ChatGPT does not pass the Turing test. An AI researcher being able to tell whether a set of responses to prompts come from an LLM or not is more like a philosopher being able to tell from someone’s writing and conversation whether they have been trained at Harvard, Heidelberg or Hunan. That is, it is more an issue of style rather than an indication of a lack of agency. Furthermore, the Turing test is meant as a test of an AI for everyman. It is not a test where an AI has to fool a leading AI researcher. The point of the test is, after all, indistinguishability with respect to conversation for normal human purposes—and it is fair to use that as a criterion for intelligence.

In the last few months, several papers have been posted to the arXiv archive where researchers have tested run Turing tests on the latest LLMs (Jones and Bergen, 2024a, 2024b; Rathi et al., 2024; Wu et al., 2024), here the results are two-sided. The overall picture is one where subjects cannot tell apart AI from humans—54% of the time GPT-4 was judged to be human—but where subjects also tended to judge real humans to be human—they did so 67% of the time (Jones and Bergen, 2024a). So if the Turing test requires reliably judging which one is a computer, humans still can reliably judge which is human, even if they are at chance at telling AI apart from a human.

But despite coming close to passing the Turing test, we are still unconvinced of ChatGPT’s agency or intelligence. As I noted earlier, there are deep connections between intelligence and agency. Most theories of agency commit to agency requiring understanding of some sort, because actions are things that are done for reasons. Doing things for reasons requires some intelligence or understanding. There are different ways we could go here; we could look for signs of intelligence that would implicate agency.^10^ One way is to ask how artificial systems perform on tasks requiring capacities, like reasoning or abstraction, which when exercised by humans implicate mental agency of some sort. While it is true that some animals can act without advanced reasoning and abstraction abilities, so agency without certain kinds of intelligence is possible, it does not follow, generally, that one can have intelligence without agency, and, in particular, that intelligence of the sort involving abstraction and reasoning does not require agency. Given the way LLMs have been designed and trained, an investigation for artificial agency might go instead via capacities like reasoning and abstraction which would be indicative of the kind of agency which in humans we would call “mental agency.” There is no *a priori* reason why artificial systems cannot possess this kind of agency without (first) acquiring bodily agency (unlike in humans and other animals).

Many researchers are now designing new test batteries to test for ChatGPT’s reasoning capacities. Before the current LLM craze, Chollet (2019) already designed an Abstraction and Reasoning Corpus (ARC). There are more recent attempts like Mitchell and colleagues’ concept ARC battery (Moskvichev et al., 2023), involving visual logic puzzles, which are intended to be easier than Chollet’s ARC so that improvements in machine capabilities could still be captured. The research on ARC benchmarks is developing rapidly and there are divergent results which are sometimes hard to interpret: some researchers report rapid one-shot learning capacities, while others deny robust abstraction (see, e.g., Kojima et al., 2022; Moskvichev et al., 2023; Xu et al., 2023; Bober-Irizar and Banerjee, 2024; Lee et al., 2024; Wu et al., 2025). There is now an ARC Prize worth more than a million US dollars for reaching the ARC benchmark^11^ and future developments are to be expected. For now, ChatGPT has some difficulty with the Concept ARC battery and we still do not really understand why LLMs do well on certain tests of reasoning and poor on others.

My diagnosis of why despite near indistinguishability from humans under certain conditions, researchers are still not convinced enough to accept ChatGPT’s agency or intelligence is the following: (1) Researchers recognise that the Turing test can be passed by “hacking” or “gaming”—that is human programmers explicit programming in heuristics which fool human observers into ascribing agency while the heuristics clearly do not underpin intelligence—and so find ways to pass it without being really agentive or intelligent, through exploiting weaknesses in the human tendency to overattribute agency. However, while this was an issue with earlier chatbot winners of the Loebner Prize, this does not seem to apply to ChatGPT, which is more robust in its conversational responses and does not rely on “hacking” tricks to hide its conversational incompetence. Yet confidence in ChatGPT as genuinely agentive or intelligent is low because (2) researchers think that ChatGPT has not done very well on more fine-grained tests, such as the ARC and other related tests, which test something more specific than behavioural indistinguishability in conversation. And (3) without a better understanding of the underlying architecture of ChatGPT and how that is explaining the perceived agency or intelligence of the responses, they remain sceptical about ChatGPT’s agency and intelligence.

This pushes us to try to look at its architecture to try to decide whether it is genuinely agentive or intelligent. What do we find there? As noted above, ChatGPT has generative pre-trained transformer models, fine-tuned using supervised learning and reinforcement learning with human feedback. What does this architecture tell us about whether it is agentive or not? Reinforcement learning would appear to show that the system has its own goals in some sense and would thus count as agentive, since reinforcement learning architectures employ algorithms which learn to generate outputs in a way that is sensitive to the instrumental value of these outputs so that the amount of reward is optimised (Butlin, 2024).^12^ Further, we could think of the human feedback in training these systems as like human feedback in the kind of teaching involved in training children or animals. So there are learning architectures underlying ChatGPT which are not dissimilar from aspects of human and animal cognition. Is this architectural aspect enough to satisfy us that ChatGPT should be treated as an agent? Despite this, the poor performance of ChatGPT on ARC and other batteries seems to suggest that while it has impressive behavioural capacities, when we look at these behavioural capacities more carefully, through the lens of more specific reasoning tasks, we see that ChatGPT does not do very well on these abstraction and reasoning tests. What does that show? In the agentive systems that we are familiar with, such as humans and in higher animals, more general behavioural capacities are underpinned by a collection of more specific reasoning, linguistic, and other agentive capacities. High performance on the more general capacities are explained by and correlated with more specific capacities. We do not find high performance on the general capacity without also high performance on these more specific capacities. Intelligence and agency are decomposable and understandable as consisting of a hierarchy of more specific behavioural capacities. Insofar as we do not find a good decomposition in the case of ChatGPT we are both sceptical about its general capacities being genuinely agentive and also somewhat at a loss as to what underpins those capacities. We understand the training regime of the AI, but we do not quite understand how the learning architecture is translating into the capacities we see.

The analysis of the agentive situation in this case would seem to be suggestive of our current predicament with respect to the question of whether AIs can be agents. The example of ChatGPT seems to suggest that to feel confident in attributing agency, we have to be able to answer fundamental questions about the grounds of agency attribution beyond behavioural grounds. Here in moving forward to the possibility of AI agency, two things are important. On the one hand, we should not just sidestep basic challenges for AI systems and should take the challenge of demonstrating that they are agentive seriously. On the other hand, we do not want to foreclose the possibility that artificial systems can act, so we need to be openminded about the agency of AI—it might look very different from the agency of systems we have encountered so far. I suggest that future research will need to bring together architectural and behavioural requirements and also provide more fine-grained analysis of specific capacities underlying general behavioural abilities, so that we can be confident of genuine competence at some level.

## Data Availability

The original contributions presented in the study are included in the article/supplementary material, further inquiries can be directed to the corresponding author.

## Footnotes

^1^There are different ways that the central problem of action is formulated, subtleties which are relevant for the dispute between causal theories of action and their opponents (cf. Frankfurt, 1978 vs. the subtraction formulation in Velleman, 2014), but we do not need to be detained by these here.

^2^An example would be an artificial system producing an output through a process of learning as opposed to an output produced because of a glitch in the circuit. But the possibility of distinguishing between cases like that is not yet to show that the former is active in a substantial way.

^3^This way I am posing A makes clear the contrast with the standard way of formulating A.

^4^Except very occasionally, by, for example, Nietzsche.

^5^One response to some of these questions is to be sceptical that “agency” is a natural kind term. I cannot address this issue here, but merely note that there is a strong science of agency in psychology and neuroscience, which would suggest that a naturalisation of agency is possible (e.g., Jeannerod, 1997). Elsewhere, I intend to map out the different purposes with which the term “agency” has been deployed in the AI and philosophy of AI literature.

^6^For example, it is implicit in the following: “… most researchers do not pay much attention to what it is that constitutes a system as an agent. Is a Khepera robot an agent, independently of its control architecture or its body, just in virtue of its capacity to move around an arena?” (Barandiaran et al., 2009).

^7^In linking proper function and architectural properties, I am not committing to whether the notion of architectural properties is an entirely structural notion, such that any intrinsic duplicate of the architecture would duplicate function, since certain biological conceptions of structure may have an etiological component that is dependent on the system’s causal history. I thank a referee for requesting this clarification.

^8^Even if we can settle on which systems count as the paradigm systems, there are difficult issues concerning how we extrapolate from paradigm agentive systems to other systems and what criteria could license such extrapolation. I will discuss some of these issues in sections 4 and 5.

^9^There have been development of other related “stances,” such as Gergely and Csibra’s teleological stance (Gergely and Csibra, 2003; Csibra and Gergely, 2013; see Juvrud and Gredebäck, 2020 for review) where a system is judged to be taking the most efficient or optimal means to the end it appears to be directed toward.

^10^What about the attempts to directly measure agency in artificial systems via agency benchmarks (e.g., Xie et al., 2024; Wu et al., 2023)? While these researchers call their tasks agency benchmarks because they are concerned with tasks that would be agentive when performed by humans, such as planning or playing games, it is unclear how these “benchmarks” provide a more specific or even an independent grasp on LLMs being agents at all, beyond their tasks being ones that are normally described in agentive vocabulary. Thanks to the referees for raising this point.

^11^ arcprize.org

^12^There is some new philosophical literature on artificial agency of interest (e.g., Butlin, 2024; Dung, 2024). I have not discussed these here because my primary point is methodological. I intend to discuss these views elsewhere.
